# Machine learning-based metabolism-related genes signature and immune infiltration landscape in diabetic nephropathy

**DOI:** 10.3389/fendo.2022.1026938

**Published:** 2022-11-22

**Authors:** Huangjie Zhang, Jinguo Hu, Junfeng Zhu, Qinglin Li, Luo Fang

**Affiliations:** ^1^ The Cancer Hospital of the University of Chinese Academy of Sciences (Zhejiang Cancer Hospital), Institute of Basic Medicine and Cancer (IBMC), Chinese Academy of Sciences, Hangzhou, China; ^2^ Department of Pharmacy, Hangzhou Traditional Chinese Medicine (TCM) Hospital Affiliated to Zhejiang Chinese Medical University, Hangzhou, China

**Keywords:** diabetic nephropathy, metabolism-related genes, immune infiltration landscape, diagnostic biomarker, machine learning

## Abstract

**Background:**

To identify the diagnostic biomarkers of metabolism-related genes (MRGs), and investigate the association of the MRGs and immune infiltration landscape in diabetic nephropathy (DN).

**Methods:**

The transcriptome matrix was downloaded from the GEO database. R package “limma” was utilized to identify the differential expressed MRGs (DE-MRGs) of HC and DN samples. Genetic Ontology (GO) and Kyoto Encyclopedia of Genes and Genomes (KEGG) analyses of DE-MRGs were performed using “clusterProfiler” R package. WGCNA, LASSO, SVM-RFE, and RFE algorithms were employed to select the diagnostic feature biomarkers for DN. The ROC curve was used to evaluate discriminatory ability for diagnostic feature biomarkers. CIBERSORT algorithm was performed to investigate the fraction of the 22-types immune cells in HC and DN group. The correlation of diagnostic feature biomarkers and immune cells were performed *via* Spearman-rank correlation algorithm.

**Results:**

A total of 449 DE-MRGs were identified in this study. GO and KEGG pathway enrichment analysis indicated that the DE-MRGs were mainly enriched in small molecules catabolic process, purine metabolism, and carbon metabolism. *ADI1*, *PTGS2*, *DGKH*, and *POLR2B* were identified as diagnostic feature biomarkers for DN *via* WGCNA, LASSO, SVM-RFE, and RFE algorithms. The result of CIBERSORT algorithm illustrated a remarkable difference of immune cells in HC and DN group, and the diagnostic feature biomarkers were closely associated with immune cells.

**Conclusion:**

*ADI1*, *PTGS2*, *DGKH*, and *POLR2B* were identified as diagnostic feature biomarkers for DN, and associated with the immune infiltration landscape, providing a novel perspective for the future research and clinical management for DN.

## Introduction

Diabetic nephropathy (DN) is one of the most common and serious complications of diabetes mellitus (DM). With the increase of incidence, DN has become one of the most important mortality factors in diabetic patients (1). In the United States, over 50,000 patients with diabetes were treated for end-stage renal disease (ESRD) (2). In China, the incidence and prevalence of DN have also increased dramatically over the past decades. Statistics in 2016 showed that the number of patients with diabetes combined with chronic kidney disease (CKD) in China is estimated to reach 24.3 million (3). In general, the prevalence of DN is expected to increase over the next few decades, especially in developing countries, as the prevalence of diabetes increases rapidly worldwide (4, 5). Concerning medical and health care expenditure has become a huge social and economic burden to society, there is an urgent need to improve the understanding of the mechanism of DN.

The pathogenesis of DN development and progression is complex and multifactorial, involving many pathways and mediators. Among them, metabolic disorders have received increasing attention. In addition to obvious glucose metabolism disorders, renal lipid homeostasis has received increasing attention in recent years (6). Unsatisfactory glycemic management and renal lipotoxicity were identified as one of the major factors in the progression of DN (6–8). Lipotoxicity is mainly associated with dysfunctional signaling and insulin resistance in non-adipose tissues such as myocardium, pancreas, skeletal muscle, liver and kidney (9). Abnormal serum lipids and ectopic renal lipid accumulation are associated with the development of renal diseases, especially diabetic nephropathy. Excessive lipid accumulation alters cellular homeostasis and activates lipogenic and glycogengenic cellular signaling pathways. The quantity and quality of lipids are involved in renal injury associated with lipotoxicity by activating inflammation, oxidative stress, mitochondrial dysfunction, and cell death (10). Anti-lipopathic agents such as statins and PPAR agonists have initially demonstrated a role in renal protection and in reducing renal lipid accumulation (6). Given the important influence of lipid metabolism on DN, coupled with the fact that protein burden can significantly influence DN progression (11), and the obvious important influence of abnormal glucose metabolism, further study of metabolism may be the key to future DN treatment.

At present, through the application of bioinformatics and genome sequencing technology, biomarkers of disease can be better identified. In this study, a variety of bioinformatics algorithms were used to investigate the role of MRGs in the pathogenesis of DN, and four MRGs were identified as diagnostic feature biomarkers of DN. In addition, the correlation between immune cells and diagnostic biomarkers was preliminarily discussed. In conclusion, the results of this study will provide new perspectives and insights for the treatment of DN patients in the future.

## Materials and methods

### Date collection

The transcriptome matrix was downloaded from the Gene Expression Omnibus (GEO) database (http://www.ncbi.nlm.nih.gov/geo/). In this study, GSE96804 and GSE30122 were downloaded from the GEO database, and the batch effect of the transcriptome matrix was removed *via* “SVA” R package. The GSE96804 contains 20 human glomeruli control samples (unaffected portion of tumor nephrectomies) and 41 human glomeruli DN samples ([HTA-2_0] Affymetrix Human Transcriptome Array 2.0 [transcript (gene) version]). The GSE30122 contains 26 glomerulus of control kidney samples, and 9 glomeruli of DN samples ([HG-U133A_2] Affymetrix Human Genome U133A 2.0 Array). Perl scripts were conducted to extract the gene expression matrix and the probes were annotated based on the platform annotation file.

### Identification of metabolism-related genes and differential expression analysis

The metabolism-related genes (MRGs) were obtained from the Molecular Signatures Database (MSigDB) (http://software.broadinstitute.org/gsea/msigdb). “C2.cp.kegg. v7.5.1.symbols.gmt” was used as the reference genes set and a total of 739 unique MRGs were extracted from the gene matrix using the Perl scripts. “limma” R package was conducted to identify the differential expression MRGs (DE-MRGs) in HC and RA group, and the threshold was set at |Fold Change| ≥ 1 and *P*-value < 0.05.

### Weighted gene co-expression network analysis

The WGCNA package was used for weighted gene co-expression network analysis (WGCNA) in R software. Firstly, DE-MRGs with variances greater than all quartile variances were selected to perform WGCNA analysis, because the results of network module analysis are easily affected by outlier samples, it was particularly important to remove outliers before constructing the network. In this study, the inter-array correlation (IAC) between chips was used to evaluate the distribution of microarray data and outliers with significantly lower mean IAC values will be removed. The co-expression network of genes conforms to no scale distribution, in another word, it follows a power law distribution. WGCNA selected the weighted coefficients to obtain the results that most accord with the scale-free network distribution. Finally, genes and gene modules were associated with clinical information to identify key genes with potential biological significance in the network. For any genes, gene significance (GS) relative to a certain dependent variable was defined as the correlation coefficient between its expression level and the level of the dependent variable. Pearson correlation coefficient was used for continuous dependent variables. For a Module, the definition of Module Significance (MS) relative to a certain dependent variable is the correlation coefficient between its characteristic gene and the level of the dependent variable. For any gene in a module, the module membership (MM) of this gene in the module is defined as the correlation coefficient between this gene and the characteristic gene of this module.

### Diagnostic feature biomarkers screening of DE-MRGs

Multiple machine learning algorithms were utilized to identify the diagnostic feature biomarkers. The least absolute shrinkage and selection operator (LASSO) was a regression analysis algorithm and this algorithm obtained a refined model by constructing a penalty function: by finally determining that the coefficients of some indicators are zero, the LASSO algorithm achieves the purpose of reducing the set of indicators. Support vector machine (SVM) was a common discrimination method usually used for pattern recognition, classification and regression analysis. Moreover, it also was a supervised learning model in the field off machine learning. To avoid overfitting and achieve reliable accuracy, recursive feature elimination (RFE) algorithm was used to select the optimal genes from the training cohort. Therefore, SVM-RFE was performed to select the appropriate feature biomarkers. Finally, the overlapping genes of WGCNA, LASSO, RFE, and SVM-RFE was considered as diagnostic feature biomarker and the expression levels of candidate genes were further validated in the validation cohort.

### Functional enrichment analysis

Gene ontology (GO) and Kyoto Encyclopedia of Genes and Genomes (KEGG) pathway enrichment analyses were performed to identify potential functional components and pathways using “clusterProfiler” R packages (12). Gene Set Enrichment Analysis (GSEA) was utilized to enrich the KEGG terms in the HC and DN group, with *P* < 0.05 was considered as statistically significant.

### Immune infiltration landscape analysis

“CIBERSORT” R package was performed to investigate the immune infiltration landscape of HC and DN samples. The gene expression of immune cells based on “CIBERSORT R script v1.03” and the algorithm was run using the LM22 signature for 1000 permutations. Correlation analysis was performed to determine the relationships between immune cells *via* “Corrplot” R package and “ggplot2” R packages was utilized to determine the difference of immune cell between HC and DN samples.

### Correlation analysis between diagnostic feature biomarkers and immune infiltrating cells

The correlation of the 4 diagnostic feature biomarkers and immune cells were estimated *via* Spearman’s rank correlation algorithm. The correlation results were visualized in lollipop diagram *via* “ggplot2” R packages, and *P*-value < 0.05 were considered as statistically significant.

### Statistical analysis

All statistical analyses were performed using R software (version 4.1.0) and Perl scripts. Correlation analyses between the two variables were performed using the Spearman’s rank correlation algorithm and *P*-value < 0.05 was considered significantly different. Differential functions were analyzed using the Wilcoxon rank-sum test between the two groups, and statistical significance was set at *P*-value < 0.05.

## Results

### Identification of differential expressed of metabolism-related genes in DN

**Figure 1:**
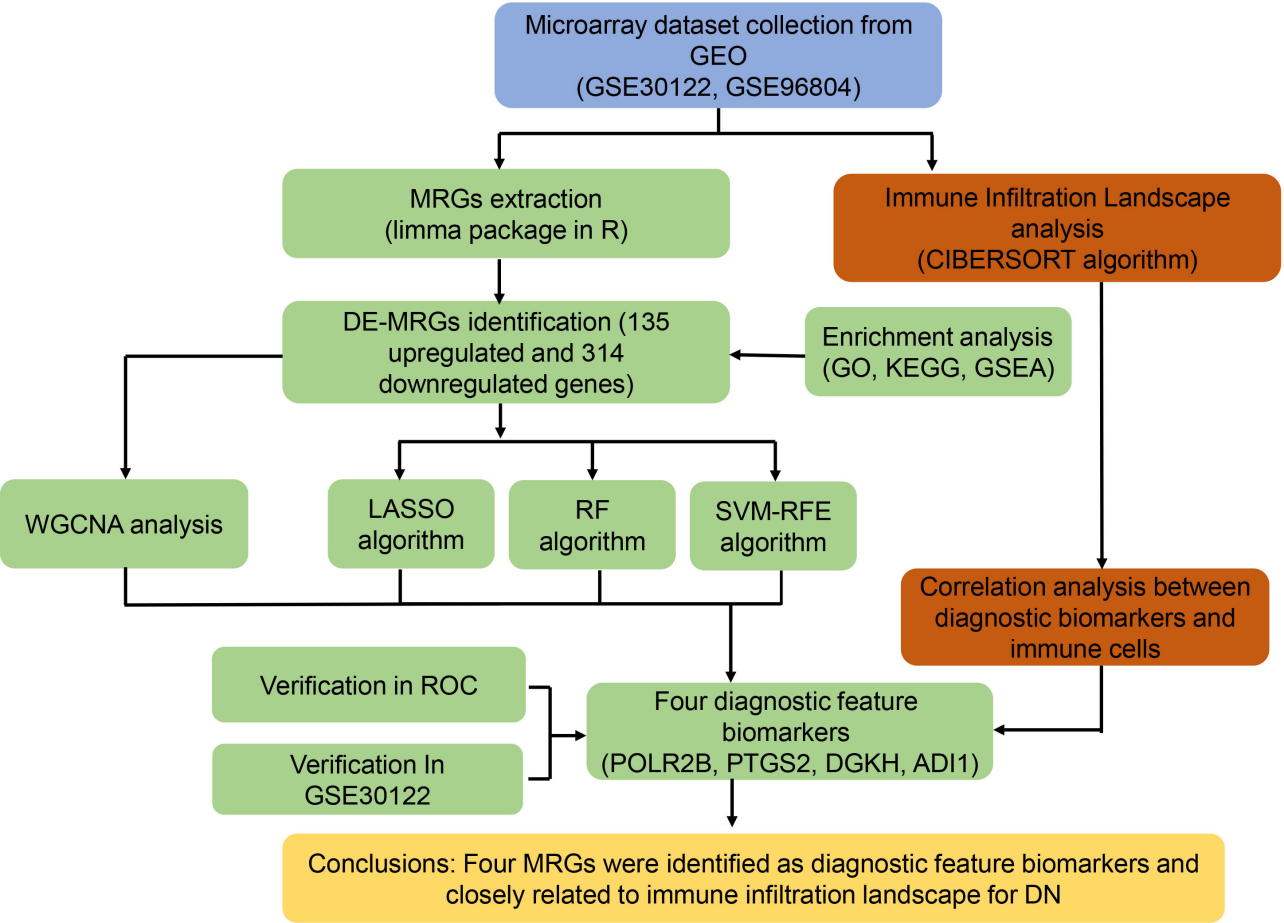
Study flow diagram.

**Figure 2:**
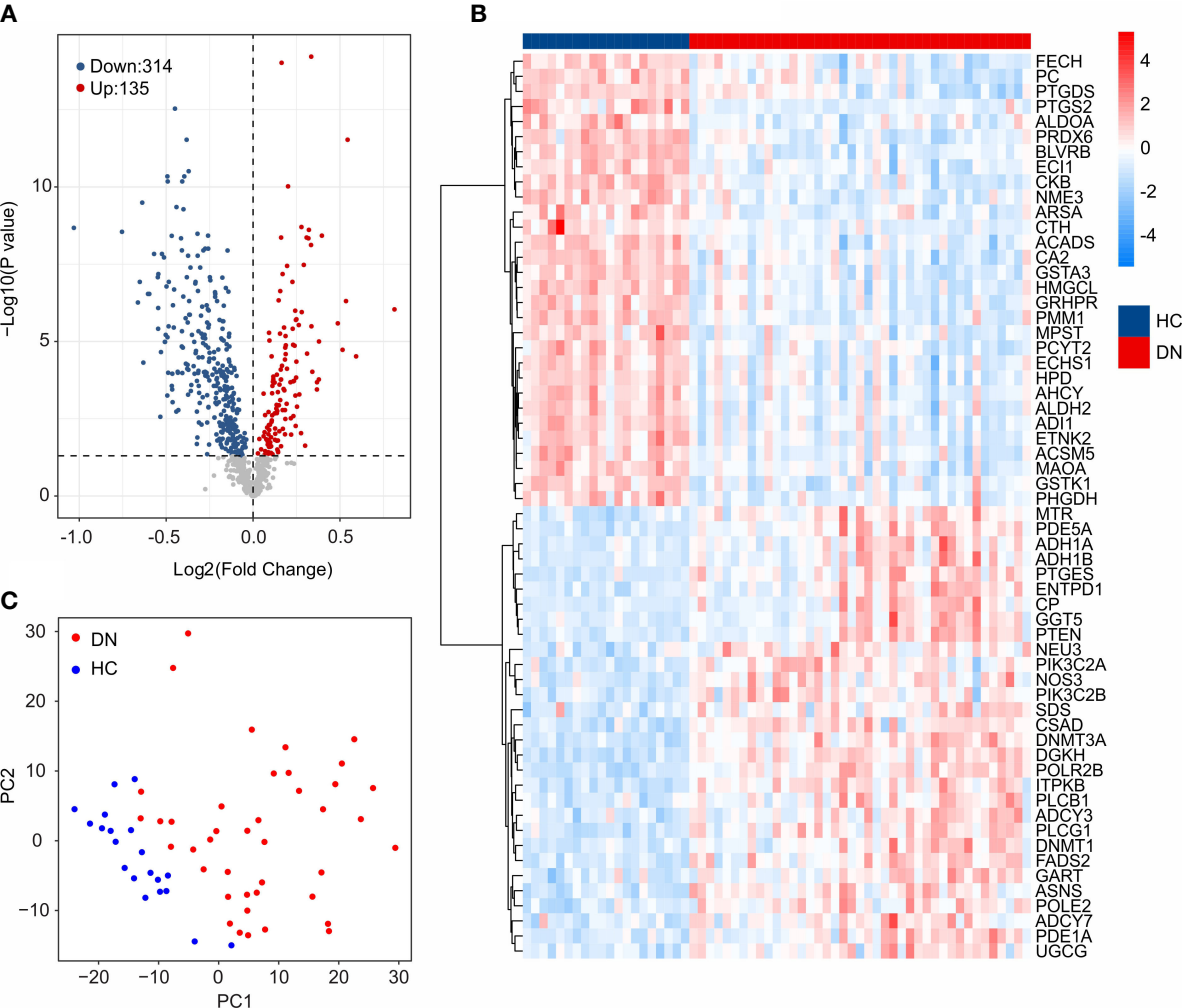
Identification of DE-MRGs. **(A)** Volcano diagram shows the DEGs in HC and DN group. The threshold for screening the DEGs is set at |Fold Change| ≥ 1 and *P* -value < 0.05. **(B)** Heatmap shows the top 30 up-regulated and down-regulated DE-MRGs in HC and DN group. **(C)** Principal component analysis illustrates a significant difference between the HC and DN samples based on the MRGs.

The workflow of this study was illustrated in the **Figure 1**. A total of 20 HC samples and 41 DN samples were extracted from the GEO database (GSE30122, GSE96804). Under the threshold set at |Fold change| ≥ 1 and *P*-value < 0.05, a total of 314 down-regulated and 135 up-regulated DE-MRGs were identified (**Figure 2A**). Heatmap diagram displayed the top 30 up-regulated and down-regulated DE-MRGs in HC and DN group (**Figure 2B**). The principal component analysis (PCA) showed a clear separation between the HC and DN group based on the MRGs (**Figure 2C**).

### Functional enrichment analysis

**Figure 3:**
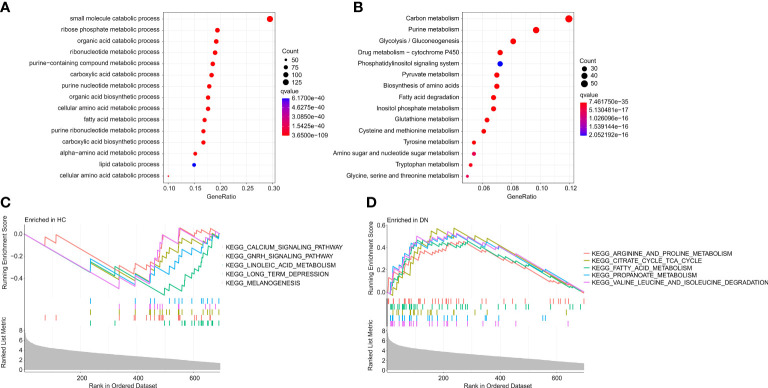
Functional enrichment analysis and GSEA analysis for DE-MRGs. **(A)** GO enrichment analysis of the DE-MRGs. **(B)** KEGG enrichment analysis shows the top 15 enriched signaling pathways of the DE-MRGs. **(C, D)** The results of GSEA displays the top 5 KEGG signaling pathways in HC and DN group.

To investigate the potential molecular mechanism of the DE-MRGs, the functional enrichment analysis was conducted. Biological process (BP) enrichment analysis showed that the DE-MRGs were significantly enriched in small molecule catabolic process, ribose phosphate metabolic process, and organic acid catabolic process (**Figure 3A**). Kyoto Encyclopedia of Genes and Genome (KEGG) enrichment analysis suggested that carbon metabolism, purine metabolism, and glycolysis/gluconeogenesis were enriched of the DE-MRGs (**Figure 3B**). GSEA results illustrated the top 5 KEGG signaling pathways in the HC and DN group. As shown in the **Figures 3C, D**, the calcium signaling pathway, GNRH signaling pathway, linoleic acid metabolism, long term depression and melanogenesis were enriched in the HC group, whereas arginine and proline metabolism, citrate cycle TCA cycle, fatty acid metabolism, propanoate metabolism, and valine leucine and isoleucine degradation were significantly enriched in the DN group.

### Identification of diagnostic feature biomarkers

**Figure 4:**
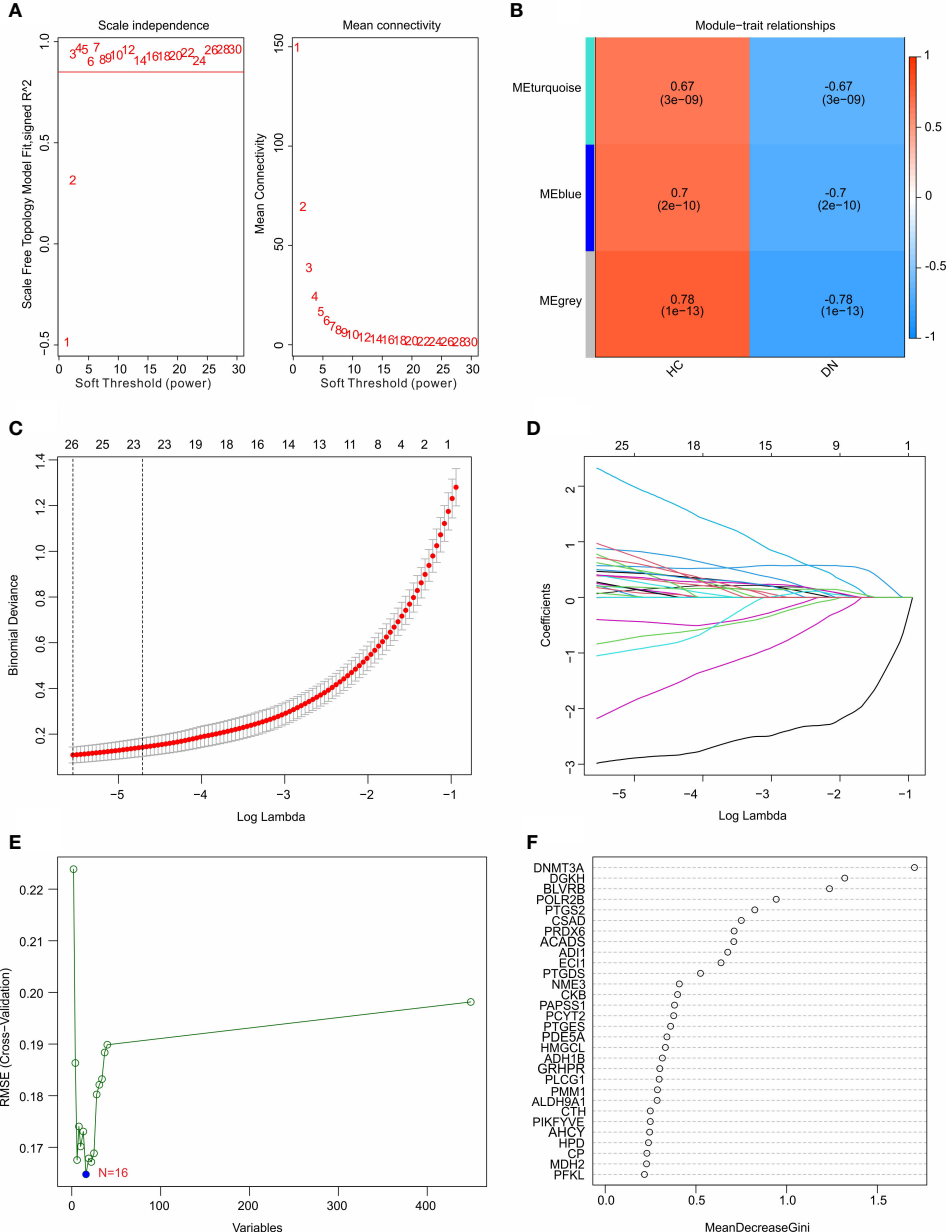
Identification of diagnostic feature biomarkers using multiple machine learning algorithm. **(A)** Analysis of the scale-free network for various soft-thresholding powers (*β*). **(B)** Heatmap shows the correlation of the module eigengenes and disease clinical characteristics. **(C, D)** Least absolute shrinkage and selection operator (LASSO) algorithm shows the optimal coefficient and minimal lambda of the DE-MRGs. **(E)** Support vector machine (SVM) algorithm shows the minimal RMSE of the DE-MRGs. **(F)** The random forest (RFE) algorithm shows the diagnostic feature biomarkers based on the DE-MRGs, with the importance more than 0.5.

Multiple machine learning algorithms were utilized to screen the diagnostic feature biomarkers in DN. A gene co-expression network was established according to weighted gene co-expression network analysis (WGCNA). The power of *β* = 6 (scale-free R^2^ > 0.85) was selected as the soft-thresholding parameter to construct a scale-free network, and the correlation coefficient of the module eigengenes and disease characteristics were calculated (**Figure 4A**). The results of the module trait showed the correlation of the module eigengenes and the disease characteristics. Of note, the module blue was positively correlated with DN, and was selected for the subsequent analysis (**Figure 4B**). Based on the LASSO algorithm, 26 DE-MRGs were identified as diagnostic feature biomarkers for DN (**Figures 4C, D**). 16 DE-MRGs were identified as diagnostic biomarkers from the DE-MRGs using the SVM-RFE algorithm (**Figure 4E**). Additionally, 11 diagnostic feature biomarkers were selected from the DE-MRGs using the RFE algorithm (**Figure 4F**).

### Identification and validation of the diagnostic feature biomarkers

**Figure 5:**
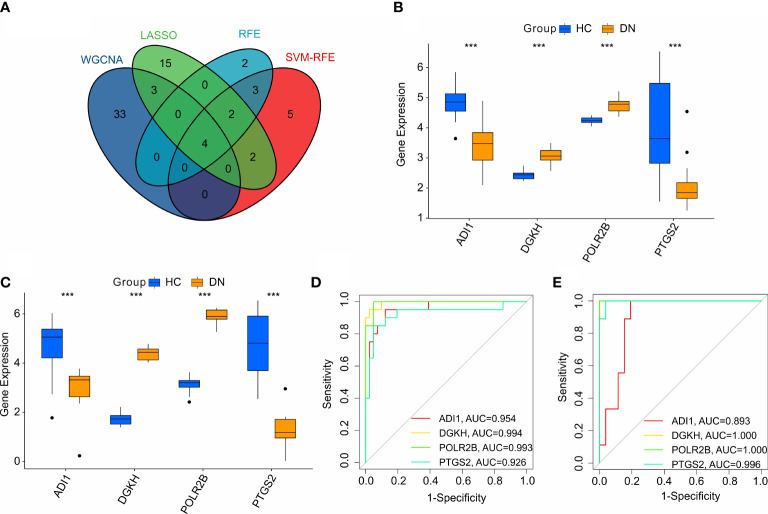
Identification and validation of the diagnostic biomarkers. **(A)** Venn diagram shows the intersection of diagnostic biomarkers based on the four machine learning algorithms. The expression of the 4 diagnostic biomarkers in training cohort **(B, C)** validation cohort. **(D, E)** ROC analysis shows the diagnostic effectiveness of the 4 diagnostic biomarkers in training cohort and validation cohort.

According to the four machine learning algorithms, 4 diagnostic feature biomarkers were identified (**Figure 5A**). The expression of the 4 diagnostic feature biomarkers in the training cohort showed that *ADI1* and *PTGS2* were expressed higher in the HC group, however, the DN group showed a higher expression of *DGKH* and *POLR2B* (**Figure 5B**). In validation cohort, the expression of *ADI1* and *PTGS2* were higher in the HC group, whereas the expression of *DGKH* and *POLR2B* were lower in the DN group compared to the HC group (**Figure 5C**). The ROC curve of the *ADI1*, *PTGS2*, *DGKH*, and *POLR2B* revealed a satisfactory diagnostic value for the 4 diagnostic feature biomarkers in the training cohort with AUC was 0.954, 0.994, 0.993, and 0.926, respectively (**Figure 5D**). The AUC of *ADI1*, *PTGS2*, *DGKH*, and *POLR2B* in the validation cohort was 0.893, 1, 1, and 0.996, respectively (**Figure 5E**). Overall, these above findings demonstrate a satisfactory diagnostic value of the 4 diagnostic feature biomarkers and could be applied to the clinical diagnosis of DN.

### Immune infiltration landscape analysis

**Figure 6:**
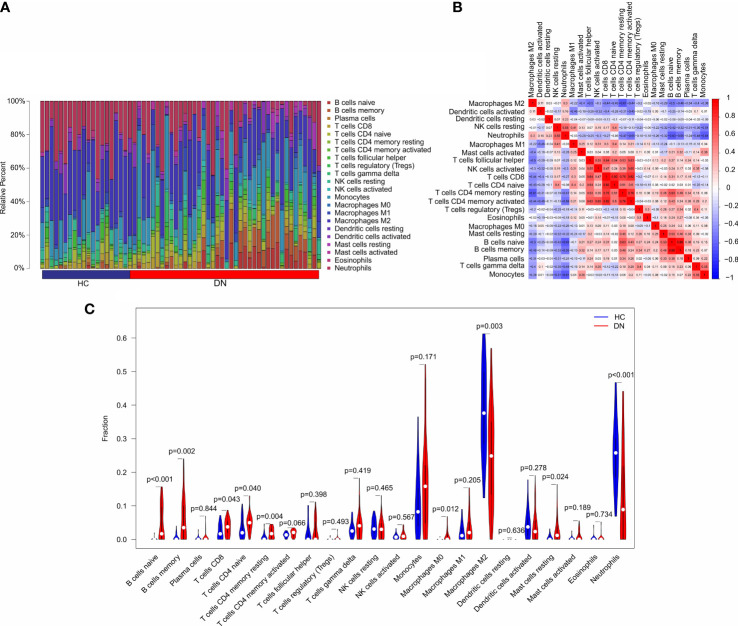
Immune infiltration analysis in HC and DN group. **(A)** Bar plot shows the fraction of the 22-types immune cells in the HC and DN group. **(B)** Correlation analysis of the 22-types immune cells. **(C)** Violin diagram shows the fraction of 22-type immune cells in HC and DN group based on the CIBERSORT algorithm.

In addition to metabolic disorders, immune system disorders are another important aspect of diabetic nephropathy (13). To investigate the immune infiltration landscape of patients, CIBERSORT algorithm was performed and the fraction of 22-types immune cells were assessed (**Figure 6A**). The correlation analysis revealed a remarkable correlation in the 22-types immune cells (**Figure 6B**). T cells CD8 was positively correlated with T cells CD4 naïve, T cells CD4 memory resting, and T cells CD4 + memory; B cells memory was positively correlated with B cells naïve; neutrophils was negatively correlated with T cells CD4 + memory, B cells naïve, B cells memory, and Monocytes; T cells CD4 memory resting was negatively correlated with macrophages M2. As shown in **Figure 6C**, the violin diagram showed that the fraction of B cells naïve, B cells memory, T cells CD8, T cells CD4 naïve, T cells CD4 memory resting, Macrophages M0, and mast cells resting were significantly higher in the DN group than HC group. However, the HC group had a higher proportion of macrophages M2 and neutrophils.

### Correlation analysis of diagnostic biomarkers and immune infiltration landscape

**Figure 7:**
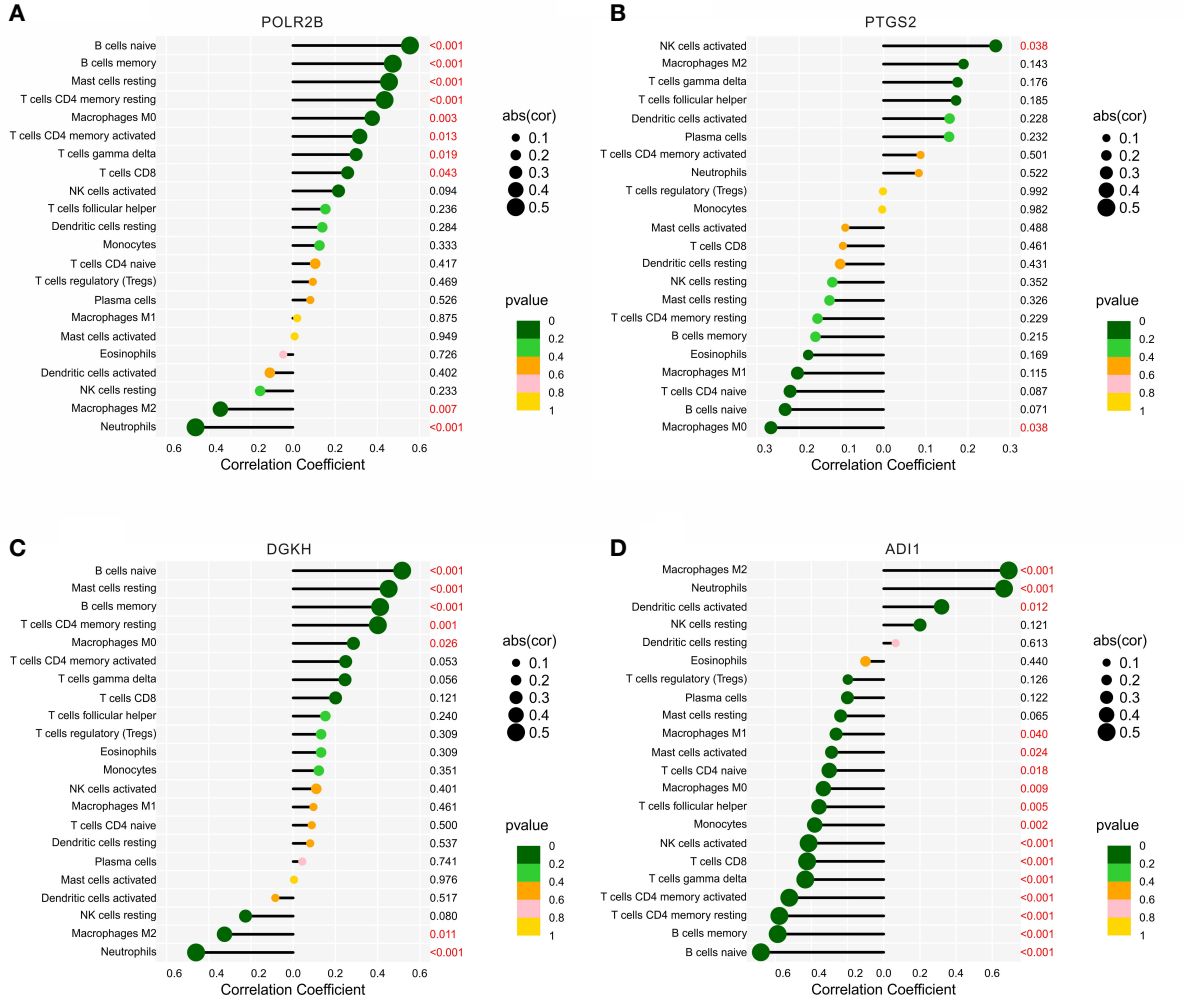
Correlation analysis between the diagnostic biomarkers and 22-types immune cells. Lollipop diagram reveals the relationship of the 22-types immune cells and **(A)** POLR2B, **(B)** PTGS2, **(C)** DGKH, and **(D)** ADI1.

The correlation analysis of the diagnostic biomarkers and immune infiltration landscape was further investigated. *POLR2B* was positively correlated with B cells naïve, B cells naïve, mast cells resting, T cells CD4 memory resting, macrophages M0, T cells CD4 memory activated, T cells gamma delta, and T cells CD8, but negatively correlated with macrophages M2 and neutrophils (**Figure 7A**). *PTGS2* was positively correlated with NK cells activated, but negatively Macrophages M0 (**Figure 7B**). *DGKH* was positively correlated with B cells naïve, Mast cells resting, B cells memory, T cells CD4 memory resting, Macrophages M0, but negatively correlated with Macrophages M2 and Neutrophils (**Figure 7C**). In addition, *ADI1* was positively correlated with macrophages M2, neutrophils, dendritic cells activated, but negatively correlated with macrophages M1, B cells memory, T cells CD4 memory resting, T cells CD4 memory activated, T cells gamma delta, T cells CD8, NK cells activated, monocytes, T cells follicular helper, macrophages M0, T cells CD4 naïve, and B cells naïve (**Figure 7D**). Taken together, these results demonstrate that the diagnostic feature biomarkers are correlated with immune infiltration landscape, providing a fresh insight for the future research in DN.

## Discussion

The involvement of metabolic diseases in the development of DN has attracted increasing attention. In this study, we used a variety of bioinformatics algorithms to study the role of MRGs in the pathogenesis of DN. Four MRGs were identified as diagnostic characteristic biomarkers of DN, indicating the importance role of MRGs in the DN process.

We observed significantly reduced *ADI1* mRNA expression levels in DN patients. As one of the four genes associated with prognosis, *ADI1* has not been reported in DN. As a metabolism-related gene, *ADI1* has been reported to be associated with the production of methionine metabolites S-adenosine methionine (SAM) and methionine sulfoxide (14). Higher plasma levels of methionine metabolites, including SAM, have been reported in diabetic nephropathy patients than in diabetic patients without nephropathy, and are associated with higher morbidity and mortality in diabetic nephropathy patients (15–17). In addition, the depletion of *ADI1* can be caused by iron depletion (18). Whereas iron deficiency has been shown to be beneficial for coronary response, endothelial dysfunction, insulin secretion, insulin action, and metabolic control in type 2 diabetes mellitus (19). In contrast, ferroptosis caused by iron overload leads to tubular cell death in diabetic nephropathy (20, 21). However, this does not explain the reduced rather than increased *ADI1* expression levels we found in DN patients. Our further data showed that *ADI1* expression level was positively correlated with M2 macrophages and neutrophils, and negatively correlated with B cells, CD4 T cells, and CD8+T cells, suggesting that *ADI1* was associated with inhibitory immunophenotype. Combined with the reduction in *ADI1* expression levels found above in DN patients, our data support the existence of reduced *ADI1* levels and increased immune system activation levels in DN patients. The involvement of the immune system as one of the important pathogenic causes of DN partially confirmed the significance of reduced *ADI1* level in DN patients.

*DGKH* diacylglycerol kinase (DGK) is a member of the enzyme family. Members of this family are involved in regulating the intracellular concentrations of diacylglycerol and phosphatidic acid. Studies on *DGKH* mainly focus on bipolar disorder, unipolar depression and adult attention-deficit/hyperactivity disorder (ADHD), etc. (22, 23). These results show that *DGKH* plays an important role in brain lipid metabolism. In addition, *DGKH* is associated with the formation of kidney stones by affecting calcium sensitive receptor (CasR) signaling and calcium metabolism in the body (24). Although there is no relevant study on *DGKH* in DN, it has been reported that there is a highly significant correlation between glomerular filtration rate, inflammation and lipid metabolism genes, supporting the possible role of abnormal lipid metabolism in the pathogenesis of DN (9). The role of *DGKH* in DN needs to be further confirmed.

As the second largest subunit encoding RNA polymerase II (Pol II), *POLR2B* catalyzes the transcription of DNA into mRNA, snRNA and microRNA precursors. This subunit and the largest subunit form opposite sides of the Pol II central cleft. Its alternative splicing results in multiple transcriptional variants (25). *POLR2B* has been reported to be associated with macular degeneration at home and abroad (26, 27). Because macular degeneration is closely related to diabetes, *POLR2B* may be involved in the process of diabetes. In addition, *POLR2B* is involved in the regulation of protein kinase, DNA-activated, catalytic subunit (PRKDC) and RNA polymerase II. The recruitment of Pol II to HBV Covalently closed circular DNA (cccDNA) assists in the phosphorylation of Pol II at Ser5 and Ser2, thereby promoting HBV transcription (28). Chronic hepatitis B virus infection as an independent predictor of renal outcome in patients with type 2 diabetes mellitus (29) also suggests an important role for *POLR2B* in DN.

Compared with *POLR2B*, *PTGS2* is convincingly involved in the development of DN. Immune dysfunction is one of the important factors of progressive nephropathy. It is traditionally believed that *PTGS2*, as an important mediator of inflammatory injury in diseases, produces prostaglandins (PGs) that are related to pathologic renal hemodynamics in diabetes and mediate renal injury caused by hemodynamic changes. (30, 31). However, recent reports suggest that the role of *PTGS2* in the development of DN and other renal injuries is complex. The expression of *PTGS2* in macrophages prevents the development of diabetic nephropathy (32). Meanwhile, specific knockdown of *PTGS2* in podocytes exacerbates diabetic nephropathy (33). This is consistent with our observation of lower M2 macrophage expression levels and lower *PTGS2* mRNA expression levels in DN. Our results support the idea that the role of *PTGS2* in renal injury may depend on the source of *PTGS2*, the mechanism of renal injury, and the expression and subtype of PGE 2 receptors (32). Further data showed *PTGS2* expression level was positively correlated with expressions of M2 macrophages, NK cells and gamma delta T cells. Meanwhile, it was negatively correlated with B cell, CD4 T cell, and CD8+ T cell expressions. Similar to ADI1, the above results also suggest that PTGS2 is associated with a suppressive immunophenotype. Combined with the decreased PTGS2 expression level we found in the above-mentioned DN patients; it is partially confirmed that the decreased *PTGS2* levels in DN patients may be related to the enhanced activation of the immune system.

The correlation between immune cells and diagnostic biomarkers was preliminarily discussed. We observed lower M2 macrophage levels in DN patients as a result of immune infiltration. In contrast to the pro-inflammatory and pro-DN effects of M1 (34), polarization of renal M1 macrophages to M2 phenotype can ameliorate experimental diabetic kidney injury by inhibiting renal M1 macrophages (34, 35). This is associated with a decrease in proinflammatory cytokines/chemokines, extracellular matrix/profibrotic proteins, and improved renal function and histology (36). In addition, PGE 2 production by *PTGS2* is important for macrophage polarization to the M2 phenotype (37). Above, we observed that lower *PTGS2* in DN may also be involved in the regulation of immune infiltration. In view of the important ameliorative effect of Pentraxin-3 and other drugs on the polarization of macrophages into M2 phenotype in DN, focusing on macrophage phenotype may play a more important role in DN treatment in the future (35).

In conclusion, the present study employed a variety of bioinformatic algorithms to investigate the role of MRGs in the pathogenesis of DN, and identified 4 MRGs as diagnostic biomarkers for DN. In addition, the correlation of immune cells with diagnostic biomarkers was initially discussed. By combining studies on the relationship between metabolism and immune microenvironment, this study provides new perspectives and insights for the future treatment of DN patients.

## Data availability statement

The raw data supporting the conclusions of this article will be made available by the authors, without undue reservation.

## Author contributions

JZ and HZ conceived and designed the study. QL contributed the data collection and data analysis. LF conceived the original ideas and composed this manuscript. JH contributed the table and figures of this manuscript. JH and HZ contributed equally to this article and all authors contributed to the article and approved the submitted version.

## Conflict of interest

The authors declare that the research was conducted in the absence of any commercial or financial relationships that could be construed as a potential conflict of interest.

## Publisher’s note

All claims expressed in this article are solely those of the authors and do not necessarily represent those of their affiliated organizations, or those of the publisher, the editors and the reviewers. Any product that may be evaluated in this article, or claim that may be made by its manufacturer, is not guaranteed or endorsed by the publisher.
